# Supplementary figures and images for: MitoTracer facilitates the identification of informative mitochondrial mutations for precise lineage reconstruction
Source: PLoS Comput Biol. 2025 Jun 23;21(6):e1013090. doi: 10.1371/journal.pcbi.1013090 (PMC12184895; doi:10.1371/journal.pcbi.1013090)

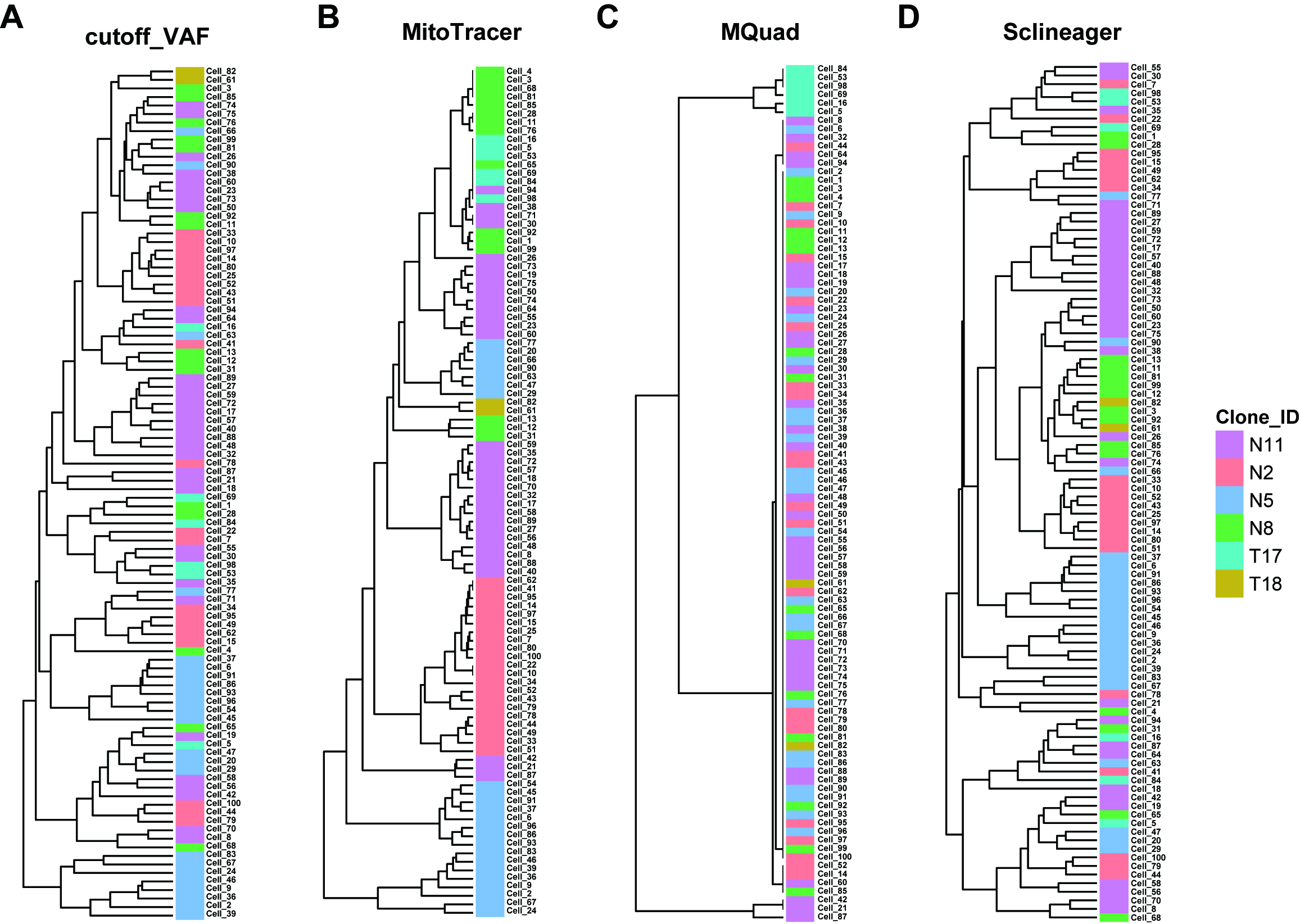

Supplement: S1 Fig — (TIF) [file pcbi.1013090.s001.tif]

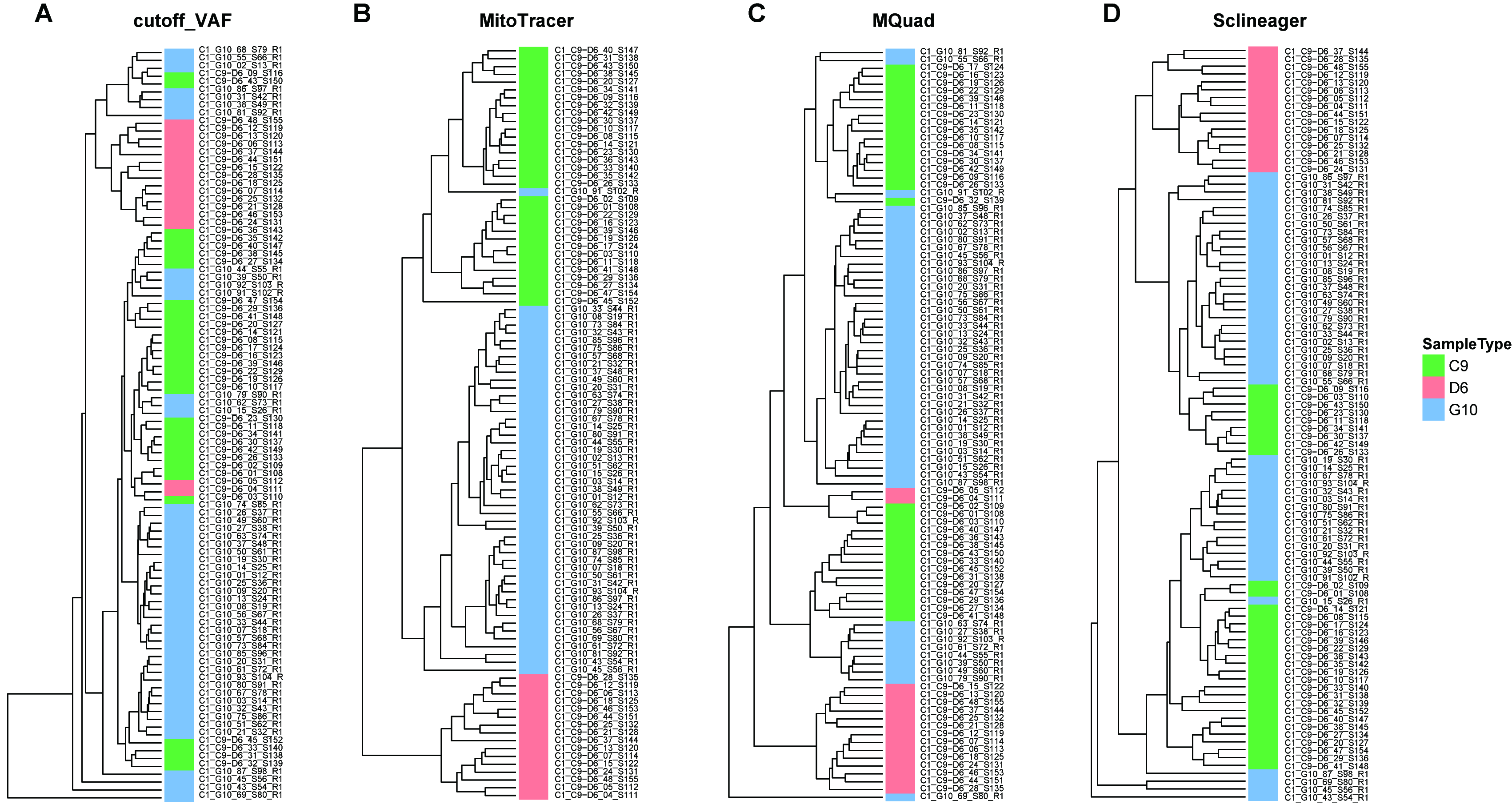

Supplement: S2 Fig — (TIF) [file pcbi.1013090.s002.tif]
